# Reply to: Reconciling differences in impact of molecular subtyping on response to cisplatin-based chemotherapy

**DOI:** 10.1038/s41467-021-24839-6

**Published:** 2021-08-10

**Authors:** Ann Taber, Emil Christensen, Philippe Lamy, Mads Agerbæk, Jørgen Bjerggaard Jensen, Lars Dyrskjøt

**Affiliations:** 1 Department of Molecular Medicine, Aarhus University Hospital, Aarhus N, Denmark; 2 Department of Clinical Medicine, Aarhus University, Aarhus C, Denmark; 3 Department of Oncology, Aarhus University Hospital, Aarhus N, Denmark; 4 Department of Urology, Aarhus University Hospital, Aarhus N, Denmark

**Keywords:** Cancer genomics, Cancer therapeutic resistance

**Replying to** Mathieu Roumiguie et al. *Nature Communications* 10.1038/s41467-021-24837-8 (2021)

Roumiguie et al., Reconciling differences in impact of molecular subtyping on response to cisplatin-based chemotherapy.

In the accompanying Comment, Roumiguie et al. correctly point out that we observe contradicting results in our study compared to earlier observations regarding *ERCC2* mutations and correlation to neoadjuvant chemotherapy (NAC) response, and the NAC benefit in tumors with a basal/squamous gene expression subtype. We thank Roumiguie et al. for the interest in our work and welcome their insights and the discussion. Here we address key factors described in the Comment regarding the discrepancies.

Roumiguie et al. suggest that some of the differences between our report and previous studies may be explained by cohort differences, as previous studies primarily focused on localized muscle-invasive bladder cancer. In our study, we included analysis of localized tumors from patients that received NAC and from patients that received first-line chemotherapy for metastatic disease. The response was defined by noninvasive pathological downstaging for patients treated with NAC and complete or partial response (RECIST 1.1) assessed by radiological imaging for patients treated with first-line chemotherapy. Both response measures evaluate tumor shrinkage following chemotherapy and therefore give a direct indication of response to cisplatin-based chemotherapy despite large differences in survival for the two patient cohorts. A complete transurethral resection, however, remains a bias for NAC response evaluation as pointed out by Roumiguie et al. Therefore, we believe it is a strength to our study that we have included patients treated with first-line chemotherapy, where the response measurements are not affected by previous surgery, and we measure a direct effect of the treatment. As illustrated in Fig. 7d in our paper, we do in fact also observe that many of the correlations to chemotherapy response show similar trends in NAC and first-line settings. However, we acknowledge that the tumor biology may have evolved and become more complex in the metastatic setting compared to the primary tumor and that site-specific circumstances associated with the metastatic process may also affect chemotherapy efficacy. Importantly, in most cases in clinical practice, tissue from metastatic sites is, however, not accessible; therefore, we find it to be of the utmost importance to investigate if correlations between response measures and primary tumor biology can be used in a metastatic setting.

Roumiguie et al. also highlight that our cohort seems to be enriched for patients with response to NAC. Patients were included from a prospective study of ctDNA analysis^1^, with no selection applied besides availability of blood samples for ctDNA measurements. However, as this study focuses on biomarker discovery, any such deviation from real-world practice may not affect the reliability of the observations—actually, a study with a balanced inclusion of responders and non-responders may be optimal for identifying robust biomarkers for later validation in clinical trials.

The fact that we did not observe a significant correlation between *ERCC2* mutations and chemotherapy response in our study was indeed puzzling—as this has been observed previously in several studies^2,3^, and in functional in vitro assays^4^. Importantly, in our NAC cohort, we did not observe *ERCC2* mutations to be significantly associated with response either^1^. This may point to the fact that a high level of tumor heterogeneity exists^5^, and that other mechanisms may be equally important^1^. However, we did observe that patients with tumors harboring somatic *BRCA2* mutations had an increased response to chemotherapy compared to *BRCA2* wild type. We observed a similar trend in TCGA data, where all patients receiving cisplatin-based chemotherapy (6/62 patients) responded. Similar observations have been presented for ovarian cancer, where *BRCA1/2*-mutated tumors showed higher response rates compared to *BRCA2* wild type^6^. Furthermore, inhibition of *BRCA2* in lung-, ovarian- and breast cancer cells have led to enhanced anti-proliferative effects of cisplatin in vitro^7^. The lack of functional *BRCA2* may provide a therapeutic opportunity, as cancer cells, not able to repair the DNA damage caused by cisplatin, are more likely to undergo apoptosis.

In addition, we would like to address the specific issues raised by Roumiguie et al. regarding the discrepancy between the Seiler et al. study^8^ and our study.

The first issue relates to the proportion of patients in the Kaplan–Meier survival analysis in Fig. 4c that received NAC treatment. The published manuscript Source Data shows that 33% (40/121) received NAC in Fig. 4c. We, however, agree that adjustment for disease state is informative and for clarity, we have included survival analysis stratified by disease state (Fig. 1). Overall survival (OS) is significantly lower for patients treated for metastatic bladder cancer and presenting with a basal/squamous gene expression subtype (*p* = 0.03). For patients treated with NAC, and where gene expression data for subtyping analysis was available, we only observed two events and no significant difference. Consequently, the observation in Fig. 4c is mainly driven by survival for patients treated for metastatic disease.

**Fig. 1:**
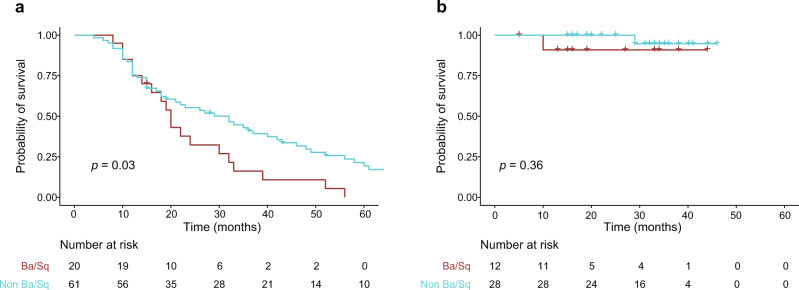
Kaplan–Meier survival analysis stratified by gene expression subtype. Probability of overall survival for patients treated with first-line chemotherapy (**a**) or NAC (**b**) with and without Ba/Sq gene expression subtype. *P* values were calculated using a log-rank test for comparing survival curves. Source data are provided as a Source data file.

Second, Roumiguie et al. raise the question whether the subtype-specific benefit of a treatment can be addressed without comparison to a non-treated cohort. On this matter, it is crucial to consider the primary endpoint of the performed analysis. We agree with Roumiguie et al. that a survival benefit should be addressed by comparing a treated to a non-treated cohort. However, for delineation of chemotherapy response biomarkers, the focus should be on treated patients with or without the biomarker in question. Importantly, the primary endpoint in our study was treatment response, and we reported on biomarkers associated with this endpoint. Nevertheless, the finding should be validated in a clinical trial setup, to assess survival benefit as highlighted by Roumigue et al. It is important to recognize that an ideal assessment of survival benefit using a non-treated control cohort will be challenging to perform, which can be exemplified by the study by Seiler et al. Here, TCGA patients that did not receive NAC represent the non-treated control cohort. However, cohort differences such as treatment modalities and inclusion criteria may complicate this cross-cohort comparison. Furthermore, according to available treatment information for TCGA (*n* = 120/412), 53% of patients in the non-NAC cohort actually received cisplatin-based chemotherapy at some time point during the disease course^7^. Therefore, the observation of better survival for patients with basal/squamous subtype tumors may be influenced by factors not related to chemotherapy response.

Third, Roumiguie et al. question the comparison between the Ba/Sq subtype and the non-Ba/Sq group consisting of the remaining subtypes. In our study, we found no significant difference in response rates when comparing all gene expression subtypes. However, as shown in Fig. 4b. we observed the lowest response rate in Ba/Sq tumors, whereas the other subtypes showed similar response rates. Based on this observation, we grouped the remaining subtypes as “non-Ba/Sq” and compared this group to Ba/Sq tumors. We acknowledge that non-Ba/Sq is a heterogenous group, and that LumP tumors are known to have more favorable prognosis in MIBC. However, the primary endpoint in our study was response to chemotherapy and not OS as discussed above.

Finally, in our study, we observed lower chemotherapy response rates for patients with a Ba/Sq gene expression subtype and for patients with low genomic instability. Interestingly, the predictive power was stronger when combined, which demonstrates the importance of investigating multiple layers of molecular data. However, based on our data, we cannot determine if it is the biology associated with the Ba/Sq subtype, the lack of genomic instability, or e.g., a lack of immune system activation in these tumors that primarily drives treatment resistance. In conclusion, the discrepancies outlined by Roumiguie et al. emphasize the need for further analysis and larger multi-omics studies to circumvent cohort bias and to enable clinical validation and ultimately delineate robust biomarkers of treatment response.

## Methods

Information regarding patients, tissue samples, nucleotide extraction procedure, and RNA-sequencing (RNA-seq) is provided in ref. ^9^. RNA-seq data were available for 121 patients (NAC: *n* = 40, first-line *n* = 81). Gene expression consensus subtypes were called as described in ref. ^9^ Cumulative survival analysis was performed using the Kaplan–Meier method, and the log-rank test was used to compare the curves (R packages survminer and survival). OS was defined as time from MIBC diagnosis to death or end of follow-up.

### Reporting summary

Further information on research design is available in the Nature Research Reporting Summary linked to this article.

## Supplementary information

Reporting Summary

## Data Availability

Raw sequencing data are deposited and available under controlled access at the European Genome-phenome Archive (EGA), which is hosted by the European Bioinformatics Institute (EBI) and the Centre for Genomic Regulation (CRG). The study accession number is EGAS00001004505. Normalized mRNA read counts are available in previously published source data^9^. Source data are provided with this paper.

## Source data

Source Data

## References

[1] Christensen E (2019). Early detection of metastatic relapse and monitoring of therapeutic efficacy by ultra-deep sequencing of plasma cell-free DNA in patients with urothelial bladder carcinoma. J. Clin. Oncol..

[2] Teo MY (2017). DNA damage response and repair gene alterations are associated with improved survival in patients with platinum-treated advanced urothelial carcinoma. Clin. Cancer Res..

[3] Van Allen EM (2014). Somatic ERCC2 mutations correlate with cisplatin sensitivity in muscle-invasive urothelial carcinoma. Cancer Discov..

[4] Li Q (2019). ERCC2 helicase domain mutations confer nucleotide excision repair deficiency and drive cisplatin sensitivity in muscle-invasive bladder cancer. Clin. Cancer Res..

[5] Meeks JJ (2020). Genomic heterogeneity in bladder cancer: challenges and possible solutions to improve outcomes. Nat. Rev. Urol..

[6] Yang D (2011). Association of BRCA1 and BRCA2 mutations with survival, chemotherapy sensitivity, and gene mutator phenotype in patients with ovarian cancer. JAMA.

[7] Rytelewski M (2014). BRCA2 inhibition enhances cisplatin-mediated alterations in tumor cell proliferation, metabolism, and metastasis. Mol. Oncol..

[8] Seiler R (2017). Impact of molecular subtypes in muscle-invasive bladder cancer on predicting response and survival after neoadjuvant chemotherapy. Eur. Urol..

[9] Taber A (2020). Molecular correlates of cisplatin-based chemotherapy response in muscle invasive bladder cancer by integrated multi-omics analysis. Nat. Commun..

