# Supplementary material for: Reply to: Reconciling differences in impact of molecular subtyping on response to cisplatin-based chemotherapy
Source: Nat Commun. 2021 Aug 10;12:4834. doi: 10.1038/s41467-021-24839-6 (PMC8355314; doi:10.1038/s41467-021-24839-6)
Supplement: Supplementary file 1 — Reporting Summary [file 41467_2021_24839_MOESM1_ESM.pdf]

nature research

Corresponding author(s): Prof. Lars Dyrskjøt

Last updated by author(s): 04-06-2021

Reporting Summary

Nature Research wishes to improve the reproducibility of the work that we publish. This form provides structure for consistency and transparency in reporting. For further information on Nature Research policies, see our Editorial Policies and the Editorial Policy Checklist.

Statistics

For all statistical analyses, confirm that the following items are present in the figure legend, table legend, main text, or Methods section.

n/a	Confirmed	
[ ]	[x]	The exact sample size (*n*) for each experimental group/condition, given as a discrete number and unit of measurement
[ ]	[x]	A statement on whether measurements were taken from distinct samples or whether the same sample was measured repeatedly
[ ]	[x]	The statistical test(s) used AND whether they are one- or two-sided<br>*Only common tests should be described solely by name; describe more complex techniques in the Methods section.*
[x]	[ ]	A description of all covariates tested
[x]	[ ]	A description of any assumptions or corrections, such as tests of normality and adjustment for multiple comparisons
[ ]	[x]	A full description of the statistical parameters including central tendency (e.g. means) or other basic estimates (e.g. regression coefficient) AND variation (e.g. standard deviation) or associated estimates of uncertainty (e.g. confidence intervals)
[ ]	[x]	For null hypothesis testing, the test statistic (e.g. *F*, *t*, *r*) with confidence intervals, effect sizes, degrees of freedom and *P* value noted<br>*Give P values as exact values whenever suitable.*
[x]	[ ]	For Bayesian analysis, information on the choice of priors and Markov chain Monte Carlo settings
[x]	[ ]	For hierarchical and complex designs, identification of the appropriate level for tests and full reporting of outcomes
[x]	[ ]	Estimates of effect sizes (e.g. Cohen's *d*, Pearson's *r*), indicating how they were calculated

Our web collection on statistics for biologists contains articles on many of the points above.

Software and code

Policy information about availability of computer code

Data collection	Study data were collected and managed using REDCap (v9.1.8) electronic data capture tools hosted at Aarhus University. REDCap (Research Electronic Data Capture) is a secure, web-based software platform designed to support data capture for research studies, providing 1) an intuitive interface for validated data capture; 2) audit trails for tracking data manipulation and export procedures; 3) automated export procedures for seamless data downloads to common statistical packages; and 4) procedures for data integration and interoperability with external sources.
Data analysis	RNA data was analyzed using the following software: Salmon v0.10.0, xCell (Web tool)

For manuscripts utilizing custom algorithms or software that are central to the research but not yet described in published literature, software must be made available to editors and reviewers. We strongly encourage code deposition in a community repository (e.g. GitHub). See the Nature Research guidelines for submitting code & software for further information.

Data

Policy information about availability of data

All manuscripts must include a data availability statement. This statement should provide the following information, where applicable:

- Accession codes, unique identifiers, or web links for publicly available datasets
- A list of figures that have associated raw data
- A description of any restrictions on data availability

Raw sequencing data are deposited and available under controlled access at the European Genome-phenome Archive (EGA), which is hosted by the European Bioinformatics Institute (EBI) and the Centre for Genomic Regulation (CRG). Study accession number is EGAS00001004505. Normalized mRNA read counts are available in previously published source data (Taber et al. 2020). The source data underlying Fig 1a-b are provided as a Source Data file.

April 2020

Field-specific reporting

Please select the one below that is the best fit for your research. If you are not sure, read the appropriate sections before making your selection.

[x] Life sciences [ ] Behavioural & social sciences [ ] Ecological, evolutionary & environmental sciences

For a reference copy of the document with all sections, see nature.com/documents/nr-reporting-summary-flat.pdf

Life sciences study design

All studies must disclose on these points even when the disclosure is negative.

Sample size	Patients were included retrospectively. No sample size calculation was performed, as all patients fulfilling the criteria necessary for a complete analysis were selected (n=300). These encompass diagnosis of muscle invasive bladder cancer followed by treatment according to national guidelines. The number of included patients proved sufficient for demonstrating significant associations between molecular correlates and clinical parameters.
Data exclusions	Patients were excluded from a multiplex platform (WES, RNA-seq, EPIC-aray or multiplex IHC) if a tumor sample were not suitable for the specific platform (low carcinoma cell fraction, low DNA or RNA concentration, fresh frozen material not available). This resulted in the partially overlapping multi-comics analyses. RNA-seq data was therefore only availeble for 121 patients.
Replication	The staining of the tissue micro arrays with individual antibodies was only performed once following optimization. We did not perform replicate experiments here, as the tissue sections are different when we cut new (tumor heterogeneity), and hence it does not make sense to make independent measurements in this way. The strength is the analysis of tissue from 184 independent patients.
Randomization	Randomization was not relevant to our study as patients were included retrospectively. In this study we included patients with bladder cancer receiving chemotherapy, focusing in cisplatin-based chemotherapy. The study design did therefore not allow for randomly assign participants into a treatment group or a control group.
Blinding	Blinding was not relevant for this retrospective study, as patients were selected to represent all response groups (RECIST V.1; complete response, partial response, stable disease and progressive disease).

Reporting for specific materials, systems and methods

We require information from authors about some types of materials, experimental systems and methods used in many studies. Here, indicate whether each material, system or method listed is relevant to your study. If you are not sure if a list item applies to your research, read the appropriate section before selecting a response.

Materials & experimental systems

n/a	Involved in the study	
[x]	[ ]	Antibodies
[x]	[ ]	Eukaryotic cell lines
[x]	[ ]	Palaeontology and archaeology
[x]	[ ]	Animals and other organisms
[ ]	[x]	Human research participants
[ ]	[x]	Clinical data
[x]	[ ]	Dual use research of concern

Methods

n/a	Involved in the study	
[x]	[ ]	ChIP-seq
[x]	[ ]	Flow cytometry
[x]	[ ]	MRI-based neuroimaging

Human research participants

Policy information about studies involving human research participants

Population characteristics	A total of 300 patients (Males = 235; Female = 65, mean±SD age at diagnosis 64 (±8)) with bladder receiving chemotherapy were included in the study; 62 received NAC before cystectomy (CX) and 245 received first-line chemotherapy upon detection of locally-advanced (T4b) or metastatic disease. Cisplatin-based chemotherapy was administered in ~98% of cases, however for the purpose of this study no distinction was made between the different chemotherapy regimens.
Recruitment	Patients were selected retrospectively based an bio bank availability. Therefore, patients were only included if they had sufficient bio-bank material to conduct at least one of our multi-omics platforms, this could have introduced a potential selection bias.
Ethics oversight	National Committee an Health Research Ethics (NVK), Denmark. (#1706291)

Note that full information on the approval of the study protocol must also be provided in the manuscript.

Clinical data

Policy information about clinical studies

All manuscripts should comply with the ICMJE guidelines for publication of clinical research and a completed CONSORT checklist must be included with all submissions.

Clinical trial registration	*Provide the trial registration number from ClinicalTrials.gov or an equivalent agency.*
Study protocol	*Note where the full trial protocol can be accessed OR if not available, explain why.*
Data collection	*Describe the settings and locales of data collection, noting the time periods of recruitment and data collection.*
Outcomes	*Describe how you pre-defined primary and secondary outcome measures and how you assessed these measures.*
